# Long-term cardiovascular complications in stage I seminoma patients

**DOI:** 10.1007/s12094-017-1742-y

**Published:** 2017-08-29

**Authors:** A. Terbuch, F. Posch, L. M. Annerer, T. Bauernhofer, M. Pichler, J. Szkandera, G. C. Hutterer, K. Pummer, R. Partl, K. S. Kapp, H. Stöger, A. Gerger, M. Stotz

**Affiliations:** 1 Division of Clinical Oncology, Department of Internal Medicine, Medical University of Graz, Auenbruggerplatz 15, 8036 Graz, Austria; 2 Research Unit Genetic Epidemiology and Pharmacogenetics, Medical University of Graz, Graz, Austria; 3 Center for Biomarker Research in Medicine (CBmed), Graz, Austria; 4 Department of Experimental Therapeutics, The University of Texas, MD Anderson Cancer Center, Houston, USA; 5 Department of Urology, Medical University of Graz, Graz, Austria; 6 Department of Therapeutic Radiology and Oncology, Medical University of Graz, Graz, Austria

**Keywords:** Testicular cancer, Seminoma, Cardiovascular risk, Radiotherapy, Carboplatin, Active surveillance

## Abstract

**Purpose:**

The cure rate of stage I seminoma patients is close to 100% and so the recent focus of clinical research has shifted onto the prevention of treatment-related complications. We assessed long-term cardiovascular complications and identified risk factors for cardiovascular events (CVEs) in stage I seminoma patients.

**Methods:**

This retrospective cohort study included 406 consecutive stage I seminoma patients. Primary endpoint was CVE rate.

**Results:**

During a median follow-up of 8.6 years, we observed 23 CVEs in 406 patients [10-year CVE risk 5.6% (95% CI 3.2 to 8.8)]. In univariable competing risk analysis, higher age, positive smoking status, history of diabetes and hypertension were significantly associated with the occurrence of CVE. In multi-state analysis, new onset of diabetes, hypertension and hyperlipidemia during follow-up predicted for an excessively increased CVE risk. In multivariable analysis adjusting for age and smoking, the development of hypertension and hyperlipidemia after tumor-specific treatment prevailed as risk factors for CVE. Regarding adjuvant treatment modalities, patients receiving adjuvant radiotherapy had a significantly higher probability of CVE than patients receiving adjuvant carboplatin [16% vs. 0%; risk difference (RD) = 16%, 95% CI 6 to 25%, *p* = 0.001]. This difference prevailed after adjusting for age, follow-up-time, diabetes, hypertension and smoking (RD = 11%, 95% CI 1 to 20%, *p* = 0.025).

**Conclusion:**

We identified a panel of baseline risk factors and dynamically, occurring predictors of CVE in stage I seminoma patients. This information may be used for targeting comorbidity management in these patients. The observed association of adjuvant radiotherapy with higher CVE risk warrants further investigation.

## Introduction

Seminoma constitutes 40% of all testicular cancers and about 80 to 85% of patients are diagnosed with clinical stage I (CS I) disease. Active surveillance, adjuvant chemotherapy with one single cycle of carboplatin (at a dose of 7 × area under curve) or adjuvant radiotherapy to paraaortic lymph nodes are treatment options for stage I seminoma [1, 2]. Regardless of treatment strategy, the cure rate is close to 100% even in case of relapse and so the recent focus of clinical research has shifted onto the prevention of treatment-related complications [3–5].

Radiotherapy and chemotherapy have both been associated with an increased risk of long-term cardiovascular complications when compared with the general population [6–10]. Chemotherapy causes endothelial damage and might, therefore, increase the risk of cardiovascular events [11–13]. In case of radiotherapy, it has been suggested that cardiac toxicity might be related to renal hypertension/diabetes mellitus secondary to partial kidney/pancreatic irradiation by the para-aortic field [6, 14]. In this retrospective cohort study, we tried to identify risk factors for the occurrence of cardiovascular complications in stage I seminoma patients and looked for differences regarding the three adjuvant treatment modalities, and potential interaction with general cardiovascular risk factors.

## Methods and patients

### Patient population

All consecutive patients (*n* = 950) with histologically confirmed TGCT, presenting to the Division of Oncology at the Medical University of Graz between January 1994 and December 2013, were retrospectively reviewed. Out of the 950 patients, 406 (44.9%) men had a tumor with seminomatous histology and CS I and were included in this retrospective cohort study. Patients were initially staged using computed tomographic (CT) scans of the abdomen, CT scan or X-ray of the chest and postoperative tumor markers α-fetoprotein (AFP), human chorionic gonadotropin (HCG) and lactate dehydrogenase (LDH). Tumor markers within normal limits after orchiectomy and the absence of metastases on imaging defined CS I. Postoperative management options were active surveillance, adjuvant radiotherapy and adjuvant single dose carboplatin. CT-based adjuvant radiotherapy to the planning target volume, which includes the paraaortic lymph nodes, was delivered using photons through opposing static fields at daily single fraction doses of 2 Gy, 5 times a week, up to a total dose of 18 to 30 Gy [15]. Follow-up data were retrieved from the database of the Division of Clinical Oncology at the Medical University of Graz until January 2015. Follow-up investigations at our center were performed according to a local protocol and were adapted in 2007 and 2012 according to recent publications [16–20]. Electronic and paper medical records of all 406 consecutive SGCT patients were retrospectively reviewed and cardiovascular events (CVEs) were documented. Cardiovascular events were defined as myocardial infarction, cerebrovascular events (stroke, transient ischemic attack) or coronary heart disease and peripheral arterial disease which had to be objectively confirmed by percutaneous coronary intervention or magnetic resonance angiography. Hyperlipidemia, hypertension and diabetes mellitus were documented when patients received treatment or when diagnosis was listed in their medical records (joint public hospital trust with common IT system and electronic healthcare database). Patient records were anonymized and de-identified prior to analysis. The study was approved by the Institutional Review Board of the Medical University of Graz (No. 26-196 ex 13/1).

### Statistical analysis

All statistical analyses were performed using STATA (Windows version 13.0, Stat Corp., Houston, TX, USA). Continuous variables, such as age, were summarized using medians (25th to 75th‰), whereas count data such as the presence of infiltration of the rete testis were reported as absolute frequencies (%). Means were compared between two or more groups using *t* tests with or without correction for heteroscedasticity as appropriate, and Kruskal–Wallis tests. The median follow-up was estimated using the inverse Kaplan–Meier method according to Schemper and Smith. The cumulative incidences of developing an arterial event were obtained with competing risk cumulative incidence estimators according to Maroubini and Valsecchi, treating death—from-any—cause as the competing event of interest. Uni- and multivariable modeling of CVE risk was performed with Fine and Gray proportional subdistribution hazards models. Due to the low event rate, we could not include a large number of predictor variables in the multivariable Fine and Gray models. Therefore, we prespecified a priori to adjust for age and smoking and, thereby, kept the number of events per predictor variable within an acceptable range. In comparing the CVE event rates between the three treatment cohorts, we could not model relative arterial event hazards because 0 patients in the adjuvant carboplatin group developed an arterial event. Instead, we directly modeled the absolute risk difference between patients receiving adjuvant carboplatin, adjuvant radiotherapy and active surveillance. Uni- and multivariable modeling of the absolute risk difference was performed using an ordinary least squares linear probability model with robust standard errors.

Missing data were present in some covariates, as reported in Table 1.

However, data were not multiply imputed, and a complete case analysis was performed. Survivor functions were analyzed with Kaplan–Meier product limit estimators, log rank tests and uni- and multivariable Cox proportional hazards models.

**Table 1:** Baseline characteristics of the patient population—distribution overall and by cardiovascular event

Variable	Subjects with available data {% missing}	Overall (*n* = 406)	CVE during follow-up (*n* = 23)	No CVE during follow-up (*n* = 383)	p*
Demographic characteristics					
Age	406 {0.0%}	37.3 [32.4 to 44.1]	46.7 [42.0 to 54.1]	37.0 [32.0 to 43.5]	0.0002
BMI	298 {26.6%}	25.3 [23.1 to 27.5]	25.3 [24.2 to 29.4]	25.2 [23.0 to 27.5]	0.353
Family history of TGCT**	282 {30.5%}	28 (9.9%)	28 (9.9%)	28 (10.3%)	0.999
Smoker or ex-smoker	329 {19.0%}	147 (44.7%)	13 (86.7%)	134 (42.7%)	0.001
Karnofsky index <100%	364 {10.3%}	17 (4.7%)	2 (12.5%)	15 (4.3%)	0.168
Diabetes pretreatment	369 {9.1%}	9 (2.4%)	5 (25.0%)	4 (1.2%)	<0.0001
Hypertension pretreatment	368 {9.4%}	20 (5.4%)	3 (16.7%)	17 (4.9%)	0.066
Hyperlipidemia pretreatment	366 {9.9%}	5 (1.4%)	0 (0.0%)	5 (1.4%)	0.990
Diabetes posttreatment	367 {9.6%}	6 (1.6%)	1 (5.9%)	5 (1.4%)	0.249
Hypertension posttreatment	368 {9.4%}	22 (6.0%)	8 (44.4%)	14 (4.0%)	<0.0001
Hyperlipidemia posttreatment	366 {9.9%}	37 (10.11%)	8 (47.1%)	29 (8.3%)	<0.0001
Clinicopathological variables					
TU size > 4 cm	352 {13.3%}	135 (38.4%)	7 (43.8%)	128 (38.1%)	0.649
Rete testis invasion	232 {43.0%}	101 (43.5%)	1 (33.3%)	100 (43.7%)	0.597
LVI	352 {13.3%}	651 (19.5%)	3 (17.7%)	62 (19.6%)	0.568
*T* stage	406 {0.0%}	/	/	/	0.277
pTis	/	2 (0.5%)	0 (00.0%)	2 (0.5%)	/
pT1	/	299 (73.7%)	14 (60.8%)	285 (74.4%)	/
pT2	/	70 (17.2%)	5 (21.7%)	65 (17.0%)	/
pT3	/	34 (8.4%)	4 (17.4%)	30 (7.8%)	/
pT4		1 (0.3%)	0 (00.0%)	1 (0.3%)	
Laboratory parameters (preoperative)					
Hemoglobin	239 {41.1%}	15.4 [14.8 to 16.3]	14.6 [14.1 to 15.4]	15.4 [14.9 to 16.4]	0.047
Leukocytes	238 {41.4%}	7.3 [5.7 to 8.8]	10.2 [7.4 to 12.0]	7.2 [5.7 to 8.7]	0.016
Thrombocytes	238 {41.4%}	225.0 [198.0 to 264.0]	230.0 [196.0 to 282.0]	224.0 [198.0 to 264.0]	0.75
CRP	209 {48.5%}	1.4 [1.0 to 3.2]	2.5 [1.4 to 8.2]	1.3 [0.9 to 3.2]	0.13
Fibrinogen	207 {49.0%}	293.0 [246.0 to 343.0]	385.0 [346.0 to 1000.0]	293.0 [246.0 to 337.0]	0.003
LDH	238 {41.4%}	199.0 [167.0 to 248.0]	218.0 [140.0 to 225.0]	198.0 [167.0 to 248.0]	0.809
Laboratory parameters (postoperative)					
CRP	195 {52.0%}	1.0 [1.0 to 2.3]	3.8 [2.3 to 4.4]	1.0 [0.9 to 2.1]	0.008
Laboratory parameters (1 year posttreatment)					
CRP	208 {48.8%}	1.0 [0.8 to 2.3]	2.8 [1.5 to 7.9]	1.0 [0.7 to 2.2]	0.008

Continuous data are reported as medians with 25th to 75th‰ in the squared brackets; categorical data are reported as absolute frequencies and percentages in parentheses. Percentages are calculated by referring only to the patients without missing values (i.e., not to the total number of patients if missing values are present)

*CVE* cardiovascular event, *BMI* body mass index, *TGCT* testicular germ cell tumor, *CRP* C-reactive protein, *LDH* lactate dehydrogenase

* *p* represents test for difference between CVE and no CVE (*χ* ^2^ tests for binary and categorical variables, ranksum-tests for continuous variables)

** Family history is defined as a history of testicular cancer in a first and/or second degree relative

To study the impact of the occurrence of intermediate events, such as hypertension, on the risk of CVEs we fitted unidirectional illness-death multistate models [21].

## Results

### Analysis at baseline

Overall, out of 950 testicular germ cell cancer patients from our in-house-research-data base, 406 patients with CS I seminoma were identified (Table 1).

Out of the 406 CS I seminoma patients, 57 (14.0%) received adjuvant radiotherapy (median dose 26 Gray), 37 (9.1%) patients received adjuvant carboplatin and 312 (76.9%) were managed with active surveillance (Table 2). Out of 57 patients treated with adjuvant radiotherapy, 1 (1.8%) experienced a relapse. In the carboplatin group, 3 (8.1%) out of 37 experienced a relapse. In the 312 patients who had chosen active surveillance, 35 (11.2%) relapsed.

**Table 2:** Baseline characteristics—distribution overall and by treatment modality

Variable	Subjects with available data {% missing}	Overall (*n* = 406)	Active surveillance (*n* = 312)	Adjuvant carboplatin (*n* = 37)	Adjuvant radiotherapy (*n* = 57)	p*
Demographic characteristics						
Age	406 {00.0%}	37.3 [32.4 to 44.1]	36.9 [32.0 to 43.1]	37.2 [31.3 to 46.1]	41.1 [34.9 to 46.5]	0.02
BMI	298 {26.6%}	25.3 [23.1 to 27.5]	25.3 [23.1 to 27.4]	24.9 [23.0 to 27.5]	25.5 [23.7 to 29.2]	0.603
Family history of TGCT**	282 {30.5%}	28 (9.9%)	22 (10.2%)	1 (3.3%)	5 (13.5%)	0.402
Smoker or ex-smoker	329 {19.0%}	147 (44.7%)	106 (42.1%)	20 (58.8%)	21 (48.8%)	0.153
Karnofsky index <100%	364 {10.3%}	17 (4.7%)	9 (3.3%)	1 (2.7%)	7 (14.0%)	0.01
Diabetes pretreatment	369 {9.1%}	9 (2.4%)	5 (1.8%)	0 (0.0%)	4 (7.4%)	0.06
Hyperlipidemia pretreatment	366 {9.9%}	5 (1.4%)	4 (1.4%)	0 (0.0%)	1 (1.9%)	0.745
Hypertension pretreatment	368 {9.4%}	20 (5.4%)	13 (4.6%)	2 (5.6%)	5 (9.6%)	0.291
Diabetes posttreatment	367 {9.6%}	6 (1.6%)	6 (2.2%)	0 (0.0%)	0 (0.0%)	0.783
Hypertension posttreatment	368 {9.4%}	22 (6.0%)	18 (6.4%)	0 (0.0%)	4 (7.7%)	0.256
Hyperlipidemia posttreatment	366 {9.9%}	37 (10.1%)	26 (9.3%)	1 (2.9%)	10 (19.2%)	0.04
Clinicopathological variables						
TU size > 4 cm	352 {13.3%}	135 (38.4%)	77 (28.6%)	26 (72.2%)	32 (68.1%)	<0.0001
Rete testis invasion	232 {43.0%}	101 (43.5%)	59 (33.9%)	19 (61.3%)	23 (85.2%)	<0.0001
Rete testis invasion and TU size > 4 cm	226 {44.3%}	40 (17.7%)	14 (8.3%)	11 (36.7%)	15 (55.6%)	<0.0001
*T* stage	406 {00.0%}	/	/	/	/	<0.277
pTis	/	2 (0.5%)	2 (0.6%)	0 (0.0%)	0 (0.0%)	/
pT1	/	299 (73.7%)	266 (85.3%)	6 (16.2%)	27 (47.4%)	/
pT2	/	70 (17.2%)	32 (10.3%)	19 (51.4%)	19 (33.3%)	/
pT3	/	34 (8.4%)	12 (3.9%)	12 (32.4%)	10 (17.5%)	/
pT4		1 (0.3%)	0 (0.0%)	0 (0.0%)	1 (1.8%)	
Laboratory parameters (preoperative)						
Hemoglobin	239 {41.1%}	15.4 [14.8 to 16.3]	15.4 [14.9 to 16.4]	15.9 [15.2 to 16.5]	15.0 [13.9 to 15.6]	0.002
Leukocytes	238 {41.4%}	7.3 [5.7 to 8.8]	7.4 [5.8 to 8.8]	7.2 [6.1 to 8.0]	6.9 [5.0 to 10.0]	0.866
Thrombocytes	238 {41.4%}	225.0 [198.0 to 264.0]	227.0 [203.0 to 264.0]	223.0 [199.0 to 271.0]	211.0 [187.0 to 249.0]	0.382
CRP	209 {48.5%}	1.4 [1.0 to 3.2]	1.3 [0.7 to 3.2]	1.1 [1.0 to 2.2]	2.4 [1.3 to 5.1]	0.022
Fibrinogen	207 {49.0%}	293.0 [246.0 to 343.0]	291.0 [245.0 to 337.0]	282.0 [248.0 to 336.0]	325.0 [288.0 to 419.0]	0.048
LDH	238 {41.4%}	199 [167 to 248]	195.0 [164.0 to 237.0]	215.0 [187.0 to 288.0]	246.0 [191.0 to 381.0]	0.002
Laboratory parameters (postoperative)						
CRP	195 {52.0%}	1.0 [1.0 to 2.3]	1.0 [0.9 to 2.3]	1.0 [0.9 to 2.3]	1.4 [1.0 to 2.1]	0.195
Laboratory parameters (1 year posttreatment)						
CRP	208 {48.8%}	1.0 [0.8 to 2.3]	1.0 [0.7 to 2.2]	1.0 [0.6 to 1.8]	1.7 [1.0 to 2.9]	0.001
Change in CRP (mg/dl) (from postoperative to 1 year posttreatment)	185 {54.4%}	0.0 [−0.4 to 0.4]	0.0 [−0.4 to 0.2]	0.0 [−0.5 to 0.5]	0.1 [−0.3 to 3.16]	0.002

Continuous data are reported as medians with 25th to 75th‰ in the squared brackets; categorical data are reported as absolute frequencies and percentages in parentheses. Percentages are calculated by referring only to the patients without missing values (i.e., not to the total number of patients if missing values are present)

*CVE* Cardiovascular event, *BMI* body mass index, *TGCT* testicular germ cell tumor, *CRP* C-reactive protein, *LDH* lactate dehydrogenase

* *p* represents test for difference between the treatment strategies

** Family history is defined as a history of testicular cancer in a first and/or second degree relative

Age at diagnosis was comparable between patients managed with active surveillance (36.9 years) and patients receiving adjuvant carboplatin (37.2 years). However, patients treated with adjuvant radiotherapy (41.1 years) were significantly older than patients on active surveillance (*p* = 0.02; Table 2).

### Analysis of arterial cardiovascular complications

During a median follow-up of 8.6 years (21 days to 21.6 years), we observed 23 arterial events. 75% of patients were followed for more than 4.4 year and 25% of patients for more than 11.2 years, respectively. Only 11 patients had a follow-up <1 year. The most frequent type of arterial event was myocardial infarction (Table 3). At the time of cardiovascular event, 2 patients (8.7%) received low dose aspirin, one patient (4.3%) received oral anticoagulation with a vitamin K antagonist, 18 patients (78.3%) did not receive any type of antithrombotic therapy and in 2 patients (8.7%) antithrombotic treatment at the time of event could not be ascertained.

**Table 3:** Overall incidence of cardiovascular events

Cardiovascular event (*N* = 23)	No. of patients (*N* = 406)	Percentage (5.7%)
Subtype of cardiovascular event		
Myocardial infarction	10	43.5
Corononary heart disease	6	26.1
Cerebrovascular event	4	17.4
Peripheral arterial disease	3	13.0

**Fig. 1:**
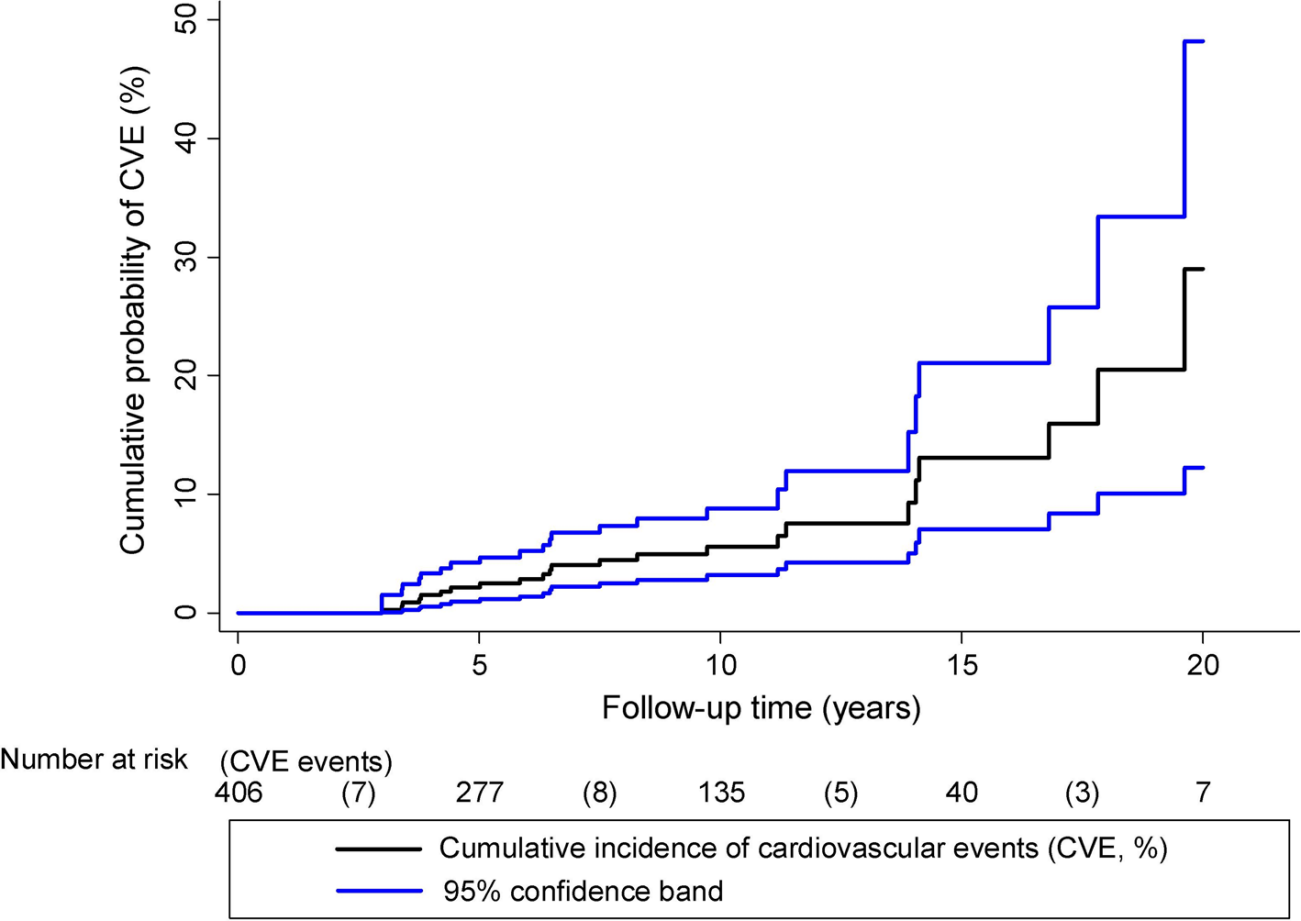
Cumulative-cardiovascular-event-risk during follow-up of testicular cancer patients

The cumulative 1, 5, 10, 15 and 20 year incidence of arterial cardiovascular events was 0.0% (95% CI 0.0 to 0.0), 2.2% (95% CI 1.0 to 4.2), 5.6% (95% CI 3.2 to 8.8), 13.1% (95% CI 7.0 to 21.1) and 29.0% (95% CI 12.2 to 48.2), respectively (Fig. 1). With 10 deaths occurring during follow-up, mortality was present as a competing risk (10-year mortality rate 3.1%, 95% CI 1.6 to 6.0).

### Predictors of cardiovascular events

In univariable competing risk analysis, higher age, positive smoking status (current or ex-smoker), history of diabetes, history of hypertension, higher preoperative leukocyte count, C-reactive protein (CRP) and fibrinogen, higher postoperative CRP and higher CRP 1 year after treatment were significantly associated with an increased risk of CVE (Table 4).

**Table 4:** Predictors of cardiovascular event risk in TGCT patients uni- and multivariable competing risk regression

Variable	Univariable HR	95% CI	*p*	Multivariable HR adjusted for age and smoking	95% CI	*p*
Demographic characteristics						
Age (per 5 years increase above 35 years)	1.62	1.35 to 1.94	<0.0001	N/A	N/A	N/A
BMI (for 5 kg/m^2^ increase above 25 kg/m^2^)	1.61	0.82 to 3.16	0.171	1.29	0.61 to 2.73	0.501
Smoker or ex-smoker	8.43	1.88 to 37.84	0.005	N/A	N/A	N/A
Karnofsky index <100%	2.65	0.70 to 9.99	0.151	2.28	0.59 to 8.87	0.233
Diabetes pretreatment	6.16	2.67 to 14.22	<0.0001	2.92	0.96 to 8.89	0.06
Hypertension pretreatment	4.84	1.37 to 17.16	0.015	1.35	0.15 to 11.86	0.79
Hyperlipidemia pretreatment	N/E	N/E	N/E	N/E	N/E	N/E
Diabetes posttreatment	16.1	1.50 to 172.70	0.022	2.62	0.19 to 35.17	0.468
Hypertension posttreatment	37.0	12.78 to 107.17	<0.0001	42.13	9.35 to 189.94	<0.0001
Hyperlipidemia posttreatment	4.12	1.49 to 11.39	0.006	3.95	1.24 to 12.62	0.021
Clinicopathological variables						
TU size > 4 cm	1.36	0.52 to 3.59	0.531	0.95	0.34 to 2.68	0.919
Rete testis invasion	0.53	0.06 to 4.87	0.575	1.01	0.08 to 11.96	0.995
Rete testis invasion plus TU size > 4 cm	1.39	0.18 to 10.98	0.755	0.60	0.07 to 4.76	0.626
Laboratory parameters (preoperative)						
Hemoglobin (per 1 g/dl increase)	0.77	0.62 to 0.96	0.020	0.88	0.62 to 1.24	0.461
Leukocytes	1.37	1.13 to 1.66	0.001	1.51	1.15 to 1.98	0.003
Thrombocytes (per 100 g/l increase)	1.77	0.58 to 5.31	0.320	2.07	0.63 to 6.83	0.233
CRP (per 10 mg/dl increase)	1.19	1.12 to 1.26	<0.0001	0.89	0.56 to 1.40	0.607
Fibrinogen (per 100 mg/dl)	1.78	1.53 to 2.07	<0.0001	1.54	1.23 to 1.94	0.0001
Preoperative LDH (per 100 U/l increase)	1.12	0.93 to 1.35	0.244	0.62	0.28 to 1.39	0.248
Laboratory parameters (postoperative)						
CRP (per 10 mg/dl increase)	2.18	1.12 to 4.24	0.022	2.31	0.85 to 6.27	0.099
Laboratory parameters (1 year posttreatment)						
CRP 1a (per 10 mg/dl increase)	23.8	4.08 to 139.21	<0.0001	6.45	0.74 to 56.22	0.092

*TGCT* testicular germ cell tumor, *CVE* cardiovascular event, *BMI* body mass index, *N/A* not applicable, *N/E* not explored due to low positive findings, *CRP* C-reactive protein, *LDH* lactate dehydrogenase

In multi-state analysis, new onset of diabetes, hypertension and hyperlipidemia during follow-up predicted for an excessively increased CVE risk.

Due to the low event rate, we could not include a large number of predictor variables in the multivariable model. However, after adjusting for age and smoking, the development of hypertension and hyperlipidemia after tumor-specific treatment prevailed as risk factors for CVE in multivariable analysis. Furthermore, inflammation markers like leukocytes and fibrinogen prevailed as risk factors. Post-treatment CRP failed to reach statistical significance (*p* 0.09) after adjustment for age and smoking, but was missing in 50% of patients (Tables 1, 4).

Out of the 23 patients who developed a CVE during follow-up, 4 patients died (median time between CVE and death = 1.9 years), and one CVE (myocardial infarction) was fatal. In a multistate model, the onset of CVE was associated with a 49-fold increase of death [transition hazard ratio (THR) = 49.0, 95 CI 10.3 to 233.0, *p* < 0.0001]. This strong association between CVE and mortality prevailed after adjusting for age (THR for CVE = 13.3, 95% CI 2.6 to 68.1, *p* 0.002).

### Adjuvant therapy and CVE risk

**Fig. 2:**
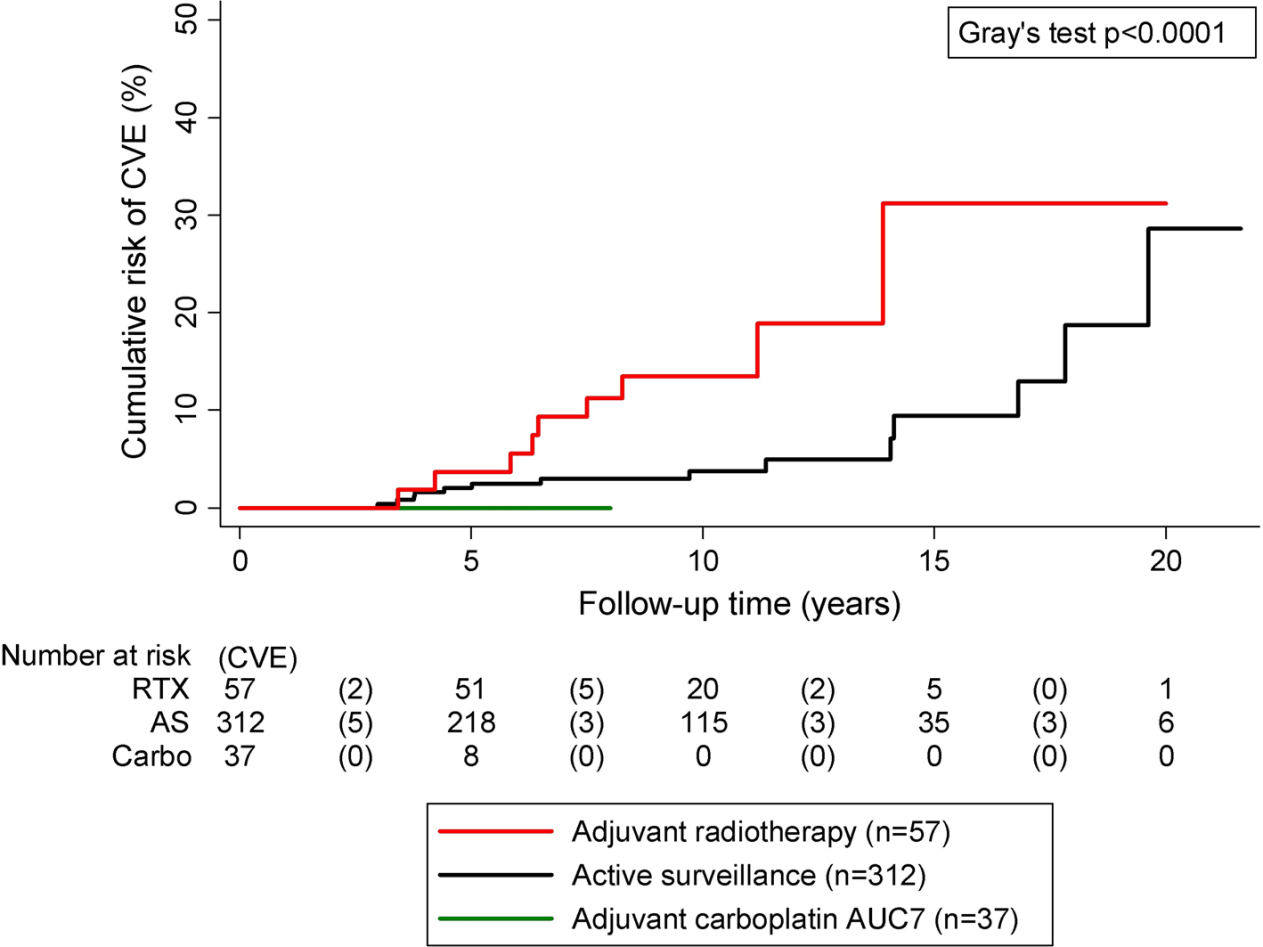
Cumulative-cardiovascular-event-risk during follow-up of testicular cancer patients depending on adjuvant therapy

During the follow-up period, we observed 9 CVEs in the 57 patients treated with adjuvant radiotherapy and 14 in the 312 patients managed with active surveillance, respectively. No CVE occurred in the 37 patients treated with single shot adjuvant carboplatin. The median follow-up was significantly longer in patients who had received adjuvant radiotherapy (9.7 years) and in patients on active surveillance (8.7 years) than in patients treated with adjuvant carboplatin (3.4 years, *p* < 0.0001). Follow-up time between patients on active surveillance and patients treated with adjuvant radiotherapy did not significantly differ (*p* 0.19). Overall, this corresponded to a 10-year-cumulative-cardiovascular-event-risk of 13.5, 3.7 and 0.0%, respectively, in these patients groups (Fig. 2). In univariable linear probability modeling, patients receiving adjuvant carboplatin had a significantly lower probability of CVE than patients on active surveillance [risk difference (RD) = −4.5%, 95% CI −6.8 to −2.2%, p < 0.0001], which can be explained by the shorter follow-up time. However, patients receiving adjuvant radiotherapy had a significantly higher probability of CVE than patients on active surveillance (RD = 11.3%, 95% CI 4.6 to 18.0%, *p* = 0.001). This difference prevailed after adjusting for age and median follow-up time (RD = 9.0%, 95% CI 2.3 to 15.8%, *p* = 0.008). Further, we observed that patients receiving adjuvant radiotherapy had a significantly higher probability of CVE than patients receiving adjuvant carboplatin (16% vs. 0%; risk difference = 16%, 95% CI 6 to 25%, *p* = 0.001). This difference also prevailed after adjusting for age, follow-up time, diabetes, hypertension and smoking (RD = 11.0%, 95% CI 1 to 20%, *p* = 0.025). These findings prompted us to explore potential mechanisms by which adjuvant radiotherapy could increase thrombotic risk. As one mechanism could be vascular inflammation in the radiation involved field, we retrospectively ascertained CRP levels 1 year after treatment. Here, we found that pretreatment CRP was higher in the radiotherapy group than in the other 2 groups, whereas this difference disappeared after surgery (Table 2), suggesting an influence of tumor inflammation because patients in the radiotherapy group had significantly higher tumor size and higher pretreatment LDH levels (Table 2). Importantly, 1 year after treatment CRP levels were significantly higher in the radiotherapy group supporting the concept of vascular inflammation post-adjuvant radiotherapy (Table 2).

## Discussion

In this retrospective cohort study, we performed a comprehensive analysis of risk factors for the occurrence of cardiovascular complication in stage I seminoma patients. Importantly, we did not only analyze baseline predictors, but also the predictive potential of new onset of hypertension, diabetes, hyperlipidemia during follow-up on long-term CVE risk. This analysis provides strong evidence that the development of components of a metabolic syndrome is strongly associated with the occurrence of cardiovascular events. Among predictors at baseline, higher age, smoking status, history of diabetes and history of hypertension were strongly associated with the development of CVE. After tumor-specific treatment new onset of diabetes, hypertension and hyperlipidemia during follow-up predicted for an excessively increased CVE risk.

Regarding adjuvant treatment modalities, patients receiving adjuvant radiotherapy had a significantly higher probability of CVE than patients receiving adjuvant carboplatin. Single dose carboplatin has been used as alternative adjuvant treatment to radiotherapy since its non-inferiority regarding relapse-free rate has been proven by the results from MRC-TE19/EORTC30982 trial in 2005 [22, 23]. Powles et al. have investigated long-term complications of 199 TGCT patients treated with single dose carboplatin and showed that there was no significant increase in cardiovascular disease when compared with age- and sex-matched general UK population, which is in line to our study results [24].

Previous studies have reported on a higher risk of cardiovascular events in TGCT patients treated with radiotherapy when compared to the general population or TGCT patients who were managed with active surveillance. For instance, Huddart et al. reported on cardiovascular morbidity of 992 TGCT survivors treated between 1982 and 1992. The risk of cardiovascular events was increased in patients treated with radiotherapy when compared to patients on active surveillance (RR = 2.4, 95% CI 1.04 to 5.45). However, in this study 8.3% of patients had received mediastinal radiotherapy, which might have biased their results [11]. Zagars et al. reported on treatment-related cardiovascular mortality in 477 men with CS I and II SGCT who received post-orchiectomy radiotherapy between 1951 and 1999. Again, 14.9% of patients had received mediastinal radiotherapy. The cardiac mortality rate was significantly elevated beyond 15 years of follow-up with standardized mortality ratio 1.95 (95% CI 1.24 to 2.94). The inclusion of patients treated with radiotherapy with higher borders for subdiaphragmatic radiotherapy fields may have resulted in larger incidental cardiovascular doses [10]. In the present study, only patients with radiotherapy to the paraaortic region have been included. Peripheral arterial disease might be explained as a result of direct vascular damage to pelvic arteries from radiotherapy. However, the most frequent cardiovascular complication in our study was myocardial infarction. This might be explained by the development of atherosclerosis through vascular inflammation. To investigate this hypothesis, we decided to look at the CRP levels 1 year after adjuvant treatment. Wethal et al. have shown that testicular cancer survivors with CRP ≥ 1.5 mg/l had 2.79 times higher risk for CVD compared to patients with CRP < 1.5 mg/l [13]. Furthermore, also an association of fibrinogen and cardiovascular disease prediction has been shown in previous studies [25, 26]. In the present study, we have retrospectively evaluated the CRP value 1 year after adjuvant treatment had finished or 1 year post-surgery if patients were managed with active surveillance. Interestingly, we found a significant difference between the 3 treatment groups. Patients in the radiotherapy group had a significantly higher CRP 1 year after treatment than patients in the carboplatin group or active surveillance group supporting the concept of vascular inflammation post-adjuvant radiotherapy. Another explanation for the increased CVE risk in TGCT survivors who have been treated with radiotherapy might be that they bear a higher risk for developing diabetes or hypertension secondary to partial pancreatic/kidney irradiation by the para-aortic field [6]. So the combination of risk factors and the resulting metabolic syndrome might be the link between radiotherapy treatment and cardiovascular complications. In our study, smoking, a history of diabetes and the development of hypertension and hyperlipidemia were significant risk factors for a cardiovascular event. In the radiotherapy group, more patients developed hyperlipidemia during follow-up compared to patients in the other 2 treatment groups. However, there was no difference between the treatment groups regarding the development of arterial hypertension and diabetes after tumor-specific treatment.

Our study has some limitations due to its retrospective nature of data collection and missing data. Furthermore, the shorter follow-up time of the patients treated with carboplatin has to be taken into account in the interpretations of our findings. The follow-up period for patients who received radiotherapy has been much longer than the follow-up of patients who received carboplatin. Both factors will increase the rate of cardiovascular late effects in the radiotherapy group. Therefore, this bias might lead to a considerable underestimation of CVE risk in the adjuvant carboplatin group. We have tried to account for this bias by adjusting for the different length of follow-up between the treatment groups. There is a considerable risk that this adjustment does not fully remove this bias. However, when comparing adjuvant radiotherapy with active surveillance, patients receiving adjuvant radiotherapy had a significantly higher probability of CVE than patients on active surveillance. Follow-up time between patients on active surveillance and patients treated with adjuvant radiotherapy did not significantly differ.

In conclusion, this study demonstrates a link between components of the metabolic syndrome at baseline and during follow-up with occurrence of long-term cardiovascular complications. The observed association of adjuvant radiotherapy with higher CVE risk warrants further prospective investigations.
